# Biological significance of GATA3, cytokeratin 20, cytokeratin 5/6 and p53 expression in muscle-invasive bladder cancer

**DOI:** 10.1371/journal.pone.0221785

**Published:** 2019-08-30

**Authors:** Chung-Chieh Wang, Yu-Chieh Tsai, Yung-Ming Jeng

**Affiliations:** 1 Department of Pathology, National Taiwan University Hospital, Taipei, Taiwan; 2 Graduate Institute of Pathology, National Taiwan University, Taipei, Taiwan; 3 Department of Oncology, National Taiwan University Hospital, Taipei, Taiwan; Centro Nacional de Investigaciones Oncologicas, SPAIN

## Abstract

Genetic profiling studies on muscle-invasive bladder cancers (MIBCs) have discovered molecular subtypes with different biological characteristics. Immunohistochemical (IHC) markers such as GATA3, cytokeratin (CK) 20, CK5/6, and p53 are associated with these subtypes. In this study, we investigated the biological and prognostic significance of these IHC markers in MIBCs from 91 patients who underwent radical cystectomy. High Ki-67 indices were associated with negative CK20 (*p* = 0.002) and diffuse CK5/6 (*p* = 0.001) staining. By contrast, tumors with diffuse GATA3 expression had low Ki-67 index (*p* = 0.006). Regarding p53, three staining patterns were associated with a high Ki-67 index: (1) complete absence, (2) diffusely strong nuclear reactivity, and (3) diffusely strong cytoplasmic staining (*p* < 0.001 compared with other patterns). CK5/6 and CK20 expression was typically present in a reciprocal fashion; however, diffuse GATA3 and CK5/6 coexpression was observed in 13 (14.29%) cases. Among 78 chemotherapy-naïve patients, low GATA3 staining was associated with worse recurrence-free survival in both univariate (*p* = 0.008) and multivariate analyses (*p* = 0.002). CK20, CK5/6, or p53 expression was not associated with clinical outcome. Based on our results, IHC staining for GATA3 may help risk stratification in patients with MIBC receiving radical cystectomy. In addition, the differences in Ki-67 indices suggested that aberrant p53 expression was better defined by the three aforementioned patterns, rather than percentage of nuclear staining alone.

## Introduction

Invasive urothelial carcinoma (UC) of the urinary bladder is a major urological and oncological disease. In the current clinical practice, advanced muscle-invasive bladder cancer (MIBC) requires aggressive management, such as radical cystectomy [1]. Immunohistochemical (IHC) markers including p53, p16, E-cadherin, and HER2/neu have been proposed for further risk stratification for MIBC [2], but none of these markers has been adapted in the current treatment guidelines [1].

In the 2010s, several studies revealed distinct molecular subtypes by analyzing mRNA expression profiles in bladder UC specimens [3–6]. For instance, Choi [3] and Damrauer [6] defined the basal and luminal subtypes of bladder UC, similar to the breast cancer molecular subtypes [7, 8]. Subsequent studies have further divided bladder UC into five [9] or six [10] subtypes and the characteristics of basal and luminal subtypes were still applied to most of the cases. In breast cancer, IHC staining for the subtype-associated markers has been incorporated in the treatment guidelines [11, 12]. Based on the achievements in breast cancer studies, surrogate IHC markers for molecular subtypes of MIBC may aid clinical management of the disease.

Choi et al [3] reported consistency between the results of IHC staining and mRNA expression profiles in basal (cytokeratin [CK] 5/6-positive) and luminal (CK20-positive) subtypes. A subsequent meta-analysis proposed that IHC study on GATA3 and CK5/6 may sufficiently identify these two subtypes with more than 90% accuracy [13]. Lerner et al [14] also defined a “basal/squamous-like” CK5/6^+^ CK14^+^ FOXA1^−^ GATA3^−^ phenotype associated with poor clinical outcome. Among these markers, negative GATA3 staining in upper-tract UC (UTUC) was associated with poor clinical outcome in one study [15] but not in another [16]. Miyamoto et al revealed poor clinical outcome in MIBC with negative or decreased GATA3 expression [17], but three other studies demonstrated no prognostic significance of GATA3 staining in bladder UC [18–20]. The importance of these IHC markers warrants further investigation.

In addition to subtype-specific genetic changes, *TP53* mutation is common in bladder UC. In the practice of diagnostic pathology, IHC expression of p53 is commonly used as a surrogate marker for *TP53* mutations. This is based on the finding that missense mutations in *TP53* increase the half-life of p53, thereby increasing the percentage of positive IHC staining for p53 [21–23]. Traditionally, a positive p53 staining is defined as nuclear reactivity over a certain cut-off percentage, whereas the absence of p53 staining indicates a negative result [24, 25]. By using this criterion, the correlation of p53 IHC staining with clinical outcome may be controversial [24]. Studies on ovarian and endometrial carcinoma have reported two patterns related to *TP53* mutation–associated aberrant p53 staining: diffusely strong nuclear reactivity and complete absence of staining [26, 27]. The cut-offs for diffuse nuclear staining were 60% and 75% for ovarian [26] and endometrial [27] carcinoma, respectively. A bladder cancer study also demonstrated that abnormal (negative or ≥50%) p53 staining was correlated with significantly worse recurrence-free survival (RFS) [25]. Therefore, the clinical significance of p53 IHC staining in bladder cancers requires further investigation.

Taken together, IHC staining for GATA3, CK20, CK5/6, and p53 may serve as surrogate tests for molecular classification and risk stratification in MIBCs; however, their association with clinical outcome warrants verification. In this study, we evaluated the IHC expression of GATA3, CK20, CK5/6 and p53 in a series of MIBC cases and correlated them with the associated clinical outcome, Ki-67 proliferative index, and other clinicopathological parameters to investigate their clinical significance.

## Materials and methods

### Patients and specimens

The patients included in this study were retrieved from the pathological diagnosis database at the Department of Pathology, National Taiwan University Hospital (NTUH). We included all 109 patients who underwent radical cystectomy for muscle-invasive bladder UC (stage T2 or higher) from 2010 to 2016 and collected their clinical data. Patients with a history of UTUC were excluded to avoid confounding prognostic analysis. In each patient who received neoadjuvant chemotherapy, the latest resection specimen prior to cystectomy was selected for this study. In this group, those who did not have a preoperative specimen available at NTUH were excluded from this study. In patients without neoadjuvant chemotherapy, the cystectomy specimens were selected. Alternatively, the latest resection specimen before cystectomy was selected if no adequate tumor cells were present in the cystectomy specimen.

Hematoxylin and eosin–stained sections of all cases were reviewed by an expert in genitourinary pathology (C.C.W.) and staged by the TNM classification system defined in the AJCC Cancer Staging Manual (8th edition). Cases without definite muscularis propria invasion were excluded at this point. One representative section with an adequate tumor part and the corresponding formalin-fixed paraffin-embedded (FFPE) tissue block were selected for each case. This study was approved by the Research Ethics Committee of NTUH on October 16, 2017 (No. 201708055RIND; revised on August 17, 2018).

### IHC staining

For each case, 5-μm sections were taken from the FFPE tissue block for IHC staining. The staining procedures were conducted with a Ventana Benchmark XT autostainer (Ventana Medical Systems, Tucson, AZ, USA) according to the manufacturer’s instructions. Primary antibodies against p53 (clone DO-7, dilution 1:1000, Dako Denmark A/S, Glostrup, Denmark), GATA3 (clone L50-823), CK20 (clone SP33), CK5/6 (clone D5/16B4), and Ki-67 (clone 30–9) were included. All antibodies other than anti-p53 antibody were purchased from Ventana Medical Systems and were ready to use. The antibody reactivity was visualised with a Ventana OptiView DAB IHC Detection Kit. Finally, the slides were counterstained with haematoxylin.

**Fig 1:**
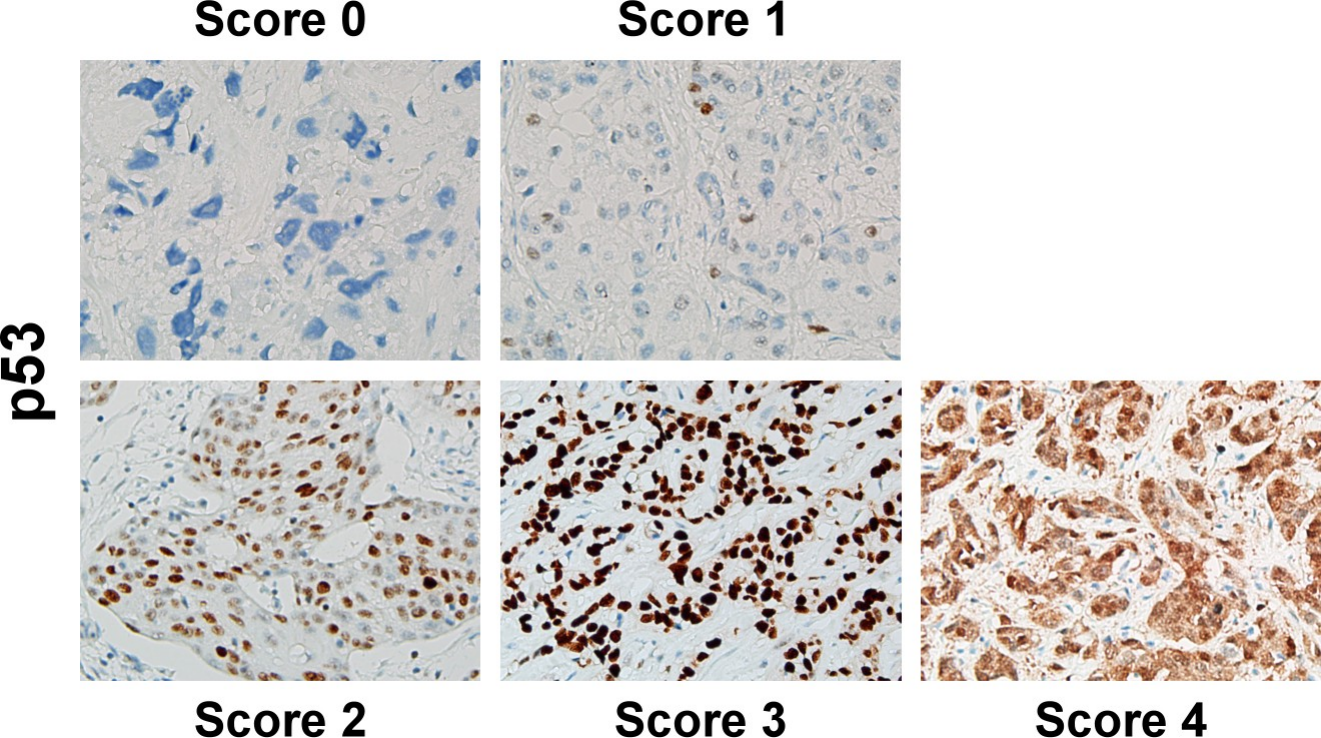
Examples of IHC staining for p53. (original magnification: ×200).

All IHC results were examined under a light microscope and scored by the same pathologist (C.C.W.) to prevent interobserver variation. The percentage and intensity of tumor cells stained with GATA3 (nuclear staining), CK20, and CK5/6 (membranous-type or cytoplasmic staining) were recorded for each case and their immunoreactive scores (IRS) were calculated using Remmele and Stegner’s criteria [28, 29] (**S1 Table**). According to the cut-offs used in IRS, their staining percentage was also classified as negative (<10%), partial (10%–80%), and diffuse (>80%). The standards for p53 scores are described in **Table 1**by combining the criteria used in prior studies of bladder [25] and ovarian [26, 30] cancers. Examples of IHC staining for these markers are displayed in **Fig 1**. The Ki-67 indices were evaluated by following the recommendations from the International Ki67 in Breast Cancer Working Group [31]. In brief, at least three 400× fields containing 500 or more invasive tumor cells were selected for each case. Tumour cells with nuclear staining were considered positive regardless of the staining intensity. The Ki-67 index was calculated as the percentage of the positive cells among the total number of tumor cells in the scored area.

**Table 1:** Scoring criteria for p53 IHC staining.

Score	Criterion
0	No nuclear staining in the tumor cells
1	Nuclear staining in 0%–50% of the tumor cells
2	Nuclear staining in ≥50% of the tumor cells with strong nuclear reactivity in <60% of the tumor cells
3	Strong nuclear staining in ≥60% of the tumor cells
4	Diffuse and strong cytoplasmic staining without nuclear pattern of score 3

### Clinicopathological correlation and survival analysis

Student *t* test and one-way analysis of variance (ANOVA) were used to evaluate differences in the percentages of IHC markers between or among comparable groups. Chi-square test and Spearman rank correlation test were applied to analyze the association among categorical and continuous parameters, respectively. In patients not receiving neoadjuvant chemotherapy, we calculated the cumulative overall survival (OS), disease-specific survival (DSS), and RFS after radical cystectomy by using the Kaplan–Meier method. The differences in survival time were determined using log-rank tests. Cox regression was used to determine the association between continuous parameters and clinical outcomes. Multivaraite analyses were also performed using Cox regression. *P* < 0.05 was considered statistically significant. Cox regression was performed with SAS (version 9.4; SAS Institute Inc., Cary, NC, USA) with the assistance of the Department of Medical Research, NTUH, and the other statistical analyses were conducted using Microsoft Excel 2007 and Prism (version 7.03; GraphPad Software, Inc., La Jolla, CA, USA).

## Results

### Demographic and clinicopathological data

Of the 109 recruited cases, definite muscularis propria invasion (i.e. stage pT2 or higher) was not identified in 9, the microscopic slides were unavailable in 7, a history of UTUC was noted in 1, and loss to follow-up after radical cystectomy was noted in 1; these 18 cases were all excluded. Finally, we included 91 patients (median age: 67 years [range: 39–89 years]; male-to-female ratio: 2.37:1). Of them, 13 (14.3%) received neoadjuvant chemotherapy. In patients not receiving neoadjuvant chemotherapy, the median follow-up time was 2.46 years. The demographic and clinicopathological data of these patients are summarized in **Table 2**, and the detailed data are available in **S1 Dataset**.

**Table 2:** Clinicopathological features of the 91 patients who received radical cystectomy.

Variable		Value
Age (year)		
	Range	39–89
	Median	67
Sex		
	Male	64 (70.3%)
	Female	27 (29.7%)
Neoadjuvant chemotherapy		
	No	78 (85.7%)
	Yes	13 (14.3%)
T stage		
	T2	30 (33.0%)
	T3	40 (44.0%)
	T4	21 (23.1%)
Lymph node metastasis		
	Absent	60 (65.9%)
	Present	31 (34.1%)
GATA3		
	Negative (<10%)	11 (12.1%)
	Partial (10%–80%)	22 (24.2%)
	Diffuse (>80%)	58 (63.7%)
CK20		
	Negative (<10%)	52 (57.1%)
	Partial (10%–80%)	21 (23.1%)
	Diffuse (>80%)	18 (19.8%)
CK5/6		
	Negative (<10%)	39 (42.9%)
	Partial (10%–80%)	19 (20.9%)
	Diffuse (>80%)	33 (36.3%)
p53 score		
	0	13 (14.3%)
	1	18 (19.8%)
	2	26 (28.6%)
	3	33 (36.3%)
	4	1 (1.1%)
Ki-67 index (%)		
	Range	5.79–96.30
	Median	56.51

### Staining for CK20 and CK5/6 was present in a reciprocal fashion

**Fig 2:**
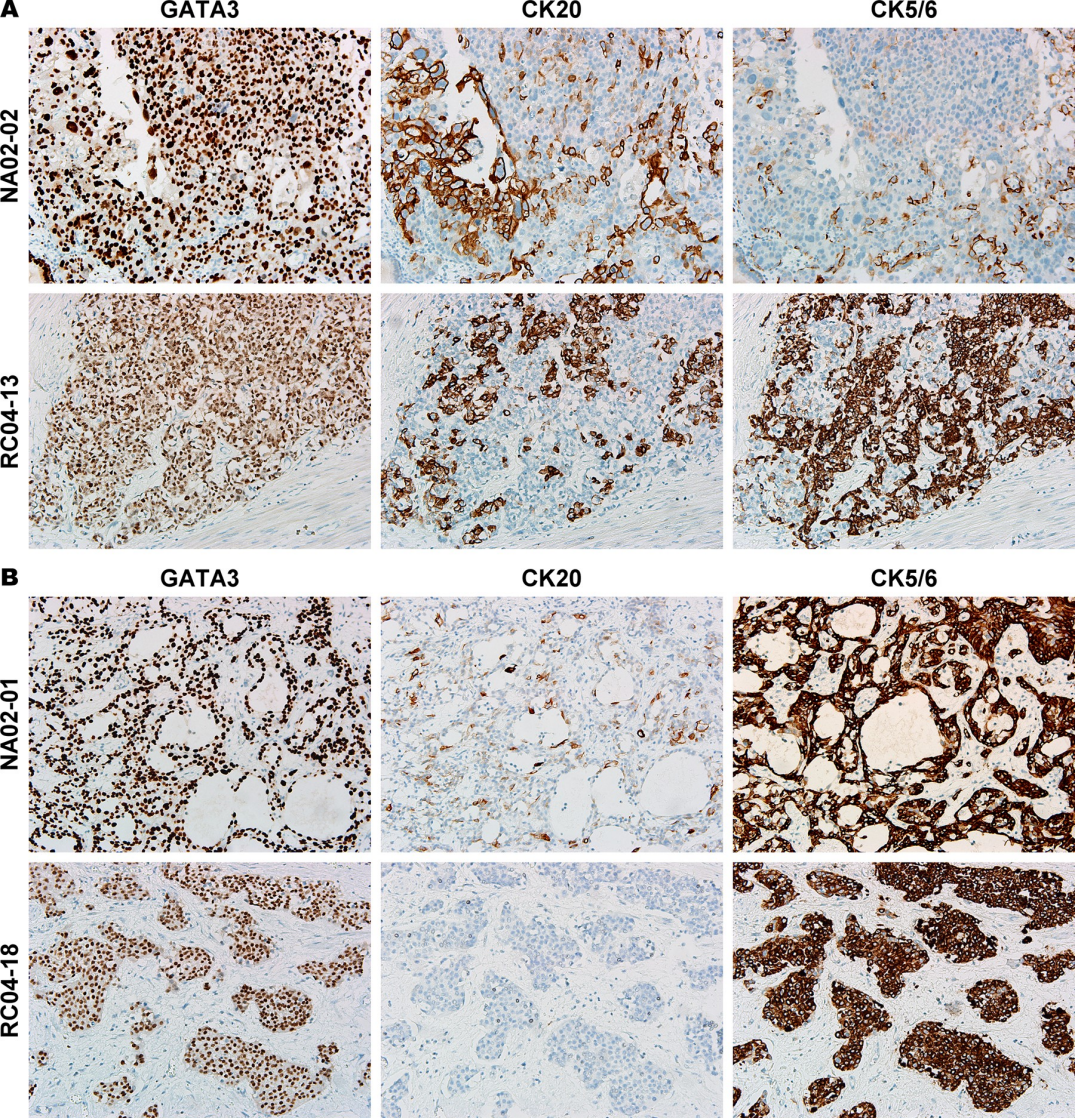
Examples of GATA3, CK20 and CK5/6 staining. (A) Two examples showing reciprocal staining patterns of CK20 and CK5/6. (B) Two examples showing diffuse coexpression of GATA3 and CK5/6.(original magnification, 200×).

After completion of IHC, we evaluated the correlation of the staining results among each marker. The results are summarized in **Table 3**as percentages and **S2 Table** as IRS. First, CK20 and GATA3 demonstrated a positive correlation in terms of both percentage and IRS score. The percentage of GATA3 staining tended to be higher than that of CK20, and CK20 staining was stained on GATA3-positive tumor cells in most cases. By contrast, a negative correlation was observed between CK20 and CK5/6. In cases with both CK20 and CK5/6 reactivity, the staining patterns of these two markers were generally reciprocal. Although they were not completely mutually-exclusive, CK20 tended to be stained on CK5/6-negative tumor cells and vice versa. **Fig 2A** illustrates two examples of such a reciprocal pattern. Of the 21 (23.08%) tumors with minimal (1%–9%) CK5/6 staining, 5 (5.49%) demonstrated basal alignment in the aggregates of tumor cells. Because the case number was limited, analyzing the biological significance of such a pattern is unfeasible.

**Table 3:** Correlation among GATA3, CK20, and CK5/6 staining percentages and Ki-67 index.

	Correlation in percentage (95% CI)			
	GATA3	CK20	CK5/6	Ki-67
GATA3	1.000	0.543 (0.375–0.677)	-0.459 (-0.611 –-0.274)	-0.321 (-0.499 –-0.117)
CK20		1.000	-0.521 (-0.660 –-0.348)	-0.377 (-0.545 –-0.179)
CK5/6			1.000	0.263 (0.054–0.450)
Ki-67				1.000

Abbreviation: CI, confidence interval

### Coexpression of GATA3 and CK5/6 was observed in a subset of MIBC

Similar to CK20, GATA3 showed negative correlation with CK5/6 expression. However, coexpression of GATA3 and CK5/6 was observed in 44 (48.35%) cases; of them 13 (14.29%) had diffuse coexpression (staining in >80% of tumor cells for both markers). Two examples of GATA3/CK5/6 coexpression are illustrated in **Fig 2B**. In the 13 double-positive cases, 11 (84.62%) showed completely absent or minimal staining for CK20. Meanwhile, double-negative tumors for GATA3 and CK5/6 accounted for 3 (3.30%) of the 91 cases, and only one of them showed complete absence for each marker.

### Aberrant p53 staining was associated with high Ki-67 indices

As presented in **Table 3**, Ki-67 indices were significantly correlated with staining percentages of GATA3, CK20, and CK5/6. We further categorized each of the latter three markers into three groups (negative, partial, and diffuse) and compared their difference through Ki-67 indices. Differences in Ki-67 indices was also compared among each group of the p53 score. The results are summarized in **Table 4**. In brief, tumors with diffuse GATA3 staining had significantly lower Ki-67 indices. By contrast, high Ki-67 indices were observed in negative CK20 and diffuse CK5/6 cases. The difference between negative and partial staining groups of GATA3 (*p* = 0.616) or CK5/6 (*p* = 0.565) was nonsignificant. Similarly, no difference was observed between cases with partial and diffuse CK20 reactivity (*p* = 0.986).

**Table 4:** Association between Ki-67 and other IHC markers.

IHC Marker			Ki-67 index (%)		
			Range	Mean ± SD	*p* value
GATA3					
	Negative or partial		15.0–96.3	63.8 ± 23.7	0.006
	Diffuse		5.8–92.0	49.5 ± 23.1	
CK20					
	Negative		5.8–96.3	61.2 ± 24.2	0.002
	Partial or diffuse		8.5–84.1	45.9 ± 21.5	
CK5/6					
	Negative or partial		5.8–95.4	48.6 ± 23.6	0.001
	Diffuse		8.5–96.3	65.3 ± 21.8	
p53					
	Each score^a^				
		0	22.3–87.9	58.9 ± 22.0	
		1	5.8–66.9	35.9 ± 19.4	
		2	8.5–82.6	47.2 ± 21.2	
		3	14.3–96.3	69.1 ± 21.1	
	0 versus 1				0.004
	1 versus 2				0.079
	2 versus 3				<0.001
	0 versus 3				0.164
	Non-aberrant versus aberrant				
		Non-aberrant (1 or 2)	5.8–82.6	42.5 ± 21.0	<0.001
		Aberrant (0, 3, or 4)	14.3–96.3	66.0 ± 21.4	

Abbreviation: SD, standard deviation

^a^Score 4 (cytoplasmic) pattern was present in only one case and not listed here.

The association between p53 score and the Ki-67 index was more complex. Absence of p53 staining (score 0) was associated with a significantly higher Ki-67 index compared with the partial staining group (score 1, *p* = 0.004). In addition, the score 2 group had a significantly lower Ki-67 index than the score 3 group (*p* < 0.001), but no difference was found between the score 1 and 2 groups (*p* = 0.079). In addition, no difference was shown between the p53-absent (score 0) and score 3 groups (*p* = 0.164). These findings were consistent with the definition of aberrant p53 expression in ovarian carcinoma [20]. Notably, 1 (1.1%) case in our cohort showed diffuse cytoplasmic staining with variable nuclear intensity (score 4). This pattern was found to be associated with *TP53* mutation in a previous study on ovarian carcinoma [28] and considered p53-aberrant. Based on this definition, tumors with aberrant p53 staining had significantly higher Ki-67 indices (*p* < 0.001). No difference in GATA3, CK20, or CK5/6 expression was noted between p53-aberrant and non-p53-aberrant tumors. Additional detailed analytical data among the IHC markers are available in **S1 Supplementary Data**.

### Intratumoral heterogeneity

In case RC01-22, a minor component (2% of total tumor area) with significantly different morphology was observed in the tumor. Although the major part showed considerable squamous differentiation, this minor component had usual histology of UC with heavy lymphocytic infiltration. As for the IHC markers, the major part expressed a typical basal/squamous profile of diffuse CK5/6 staining, minimal GATA3 reactivity, and a non-aberrant pattern of p53. By contrast, the minor component was partially positive for both GATA3 and CK5/6 with diffusely strong reactivity to p53 (**S1 Fig**). This patient demonstrated tumor recurrence after radical cystectomy, but the diagnosis of recurrence was based on radiologic images without acquisition of tissue specimens. For analytical purposes, the IHC profile of the major part was used for this case. No other tumor with apparent intratumoral heterogeneity was noted in our study cohort.

### GATA3 reactivity was significantly associated with clinical outcomes

**Fig 3:**
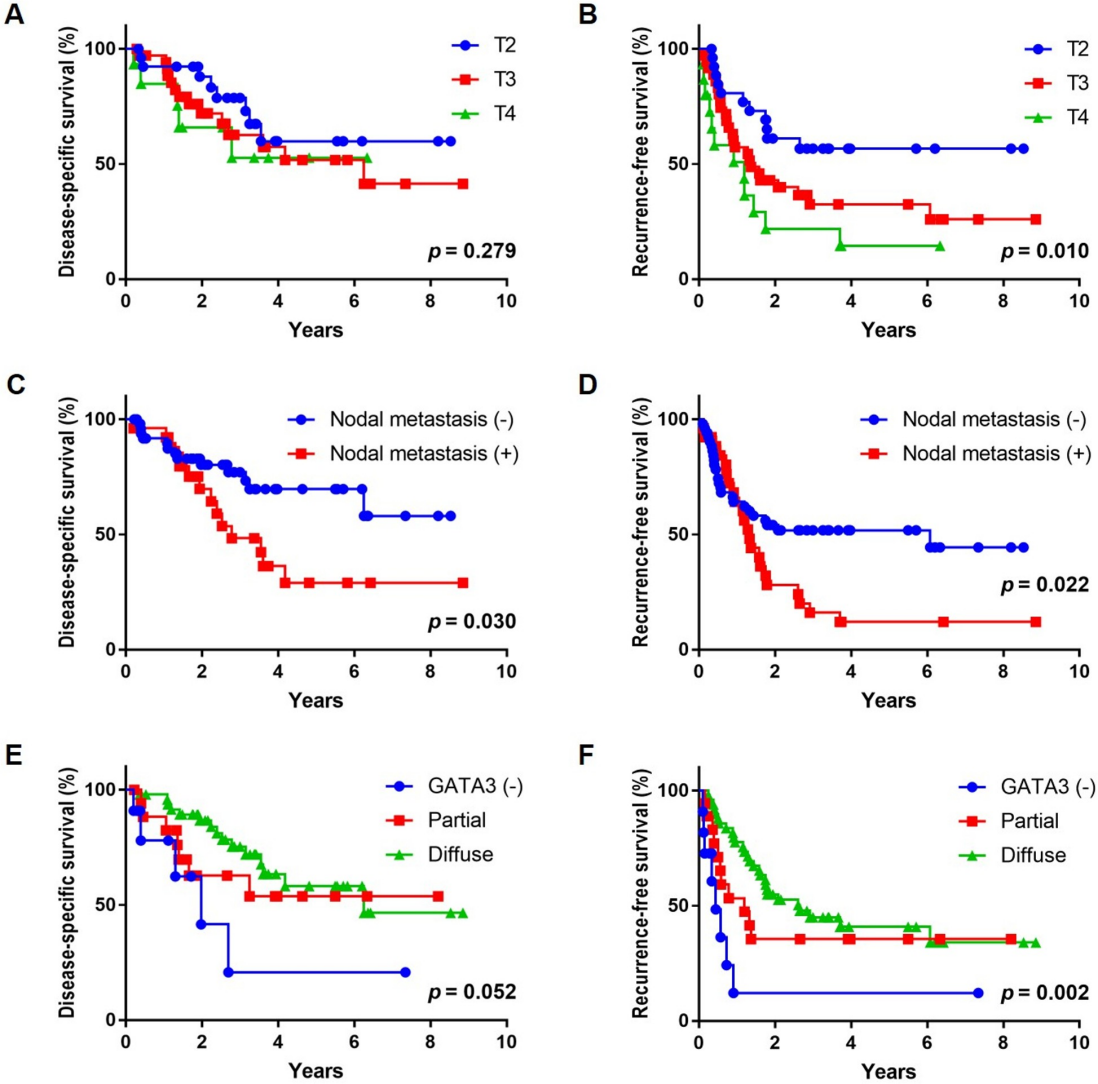
Kaplan–Meier curves regarding the 78 patients not receiving neoadjuvant chemotherapy. (A, B) DSS and RFS stratified by T stage. (C, D) DSS and RFS stratified by nodal status. (E, F) DSS and RFS stratified by GATA3 expression.

Among the IHC markers (GATA3, CK20, CK5/6, p53 and Ki-67), only GATA3 demonstrated significant correlation with clinical outcomes. In the 78 patients without neoadjuvant chemotherapy, higher percentage of GATA3 staining was associated with a significantly better RFS in both univariate (*p* = 0.008) and multivariate (*p* = 0.002) analysis by using Cox regression (**Table 5**). Analysis by IRS revealed similar results (**S3 Table**). In the Kaplan-Meier plot, gradual difference in RFS was present among cases with diffuse, partial, and negative GATA3 staining (*p* = 0.002). The other significant prognostic parameters included the T stage (for RFS) and the presence of nodal metastasis (for DSS and RFS). The Kaplan–Meier plots for GATA3, T stage, and nodal metastasis are depicted in **Fig 3**.

**Table 5:** Univariate and multivariate Cox regression analysis of clinical outcomes in 78 patients not receiving neoadjuvant chemotherapy.

Variable	Univariate		Multivariate	
	HR (95% CI)	*p* value	HR (95% CI)	*p* value
**RFS**				
T stage (T2 reference)	1.837 (1.230–2.745)	0.003	1.697 (1.083–2.658)	0.021
Nodal metastasis (N1–3 vs. N0)	1.944 (1.091–3.463)	0.024	1.821 (0.968–3.424)	0.063
GATA3 (by percentage)	0.342 (0.156–0.751)	0.008	0.270 (0.120–0.608)	0.002
CK20 (by percentage)	0.593 (0.276–1.274)	0.181		
CK5/6 (by percentage)	1.580 (0.818–3.051)	0.173		
p53 (aberrant vs. non-aberrant)	1.053 (0.594–1.866)	0.860		
Ki-67 index	0.713 (0.199–2.555)	0.603		
**DSS**				
T stage (T2 reference)	1.343 (0.786–2.294)	0.281		
Nodal metastasis (N1–3 vs. N0)	2.257 (1.059–4.811)	0.035		
GATA3 (by percentage)	0.414 (0.149–1.147)	0.090		
CK20 (by percentage)	0.597 (0.208–1.718)	0.339		
CK5/6 (by percentage)	1.443 (0.608–3.428)	0.406		
p53 (aberrant vs. non-aberrant)	1.249 (0.583–2.675)	0.567		
Ki-67 index	0.442 (0.084–2.321)	0.335		
**OS**				
T stage (T2 reference)	1.460 (0.887–2.403)	0.136		
Nodal metastasis (N1–3 vs. N0)	1.702 (0.838–3.457)	0.142		
GATA3 (by percentage)	0.484 (0.183–1.281)	0.144		
CK20 (by percentage)	0.539 (0.197–1.471)	0.227		
CK5/6 (by percentage)	1.663 (0.746–3.709)	0.214		
p53 (aberrant vs. non-aberrant)	1.214 (0.597–2.467)	0.592		
Ki-67 index	0.390 (0.083–1.824)	0.232		

Abbreviations: HR, hazard ratio; CI, confidence interval

## Discussion

GATA3 is a transcription factor useful in histological diagnosis for UC [18, 32, 33]. It is also recognized as a marker of luminal subtype in bladder cancer according to recent research [3, 9, 13]. In this study, we noted that tumors with decreased GATA3 staining had significantly higher Ki-67 proliferative indices. In addition, IHC staining for GATA3 was correlated with a clinical outcome in chemotherapy-naïve patients with MIBC. Cases with diffuse GATA3 staining had the best outcome, and a minor proportion (12.1%) of GATA3-negative tumors were prone to early recurrence with a borderline trend of worse DSS. The prognostic significance of GATA3 was independent to stage and nodal metastasis in RFS. These findings are compatible with the relatively aggressive behavior observed in the basal/squamous-like tumors [3, 6, 10, 13].

The basal/squamous-like phenotype described by Lerner et al included positive CK5/6 and negative GATA3 in IHC staining [14]. However, we found it difficult to define the actual “basal/squamous-like” subgroup in our study cohort. In this study, diffusely strong CK5/6 staining was not necessarily associated with negative GATA3. The association of these two markers with the Ki-67 index might aid in understinaing this problem. Difference in the Ki-67 index was significant at a 80% cut-off for GATA3, but the tumors with negative and partial GATA3 staining had similar Ki-67 indices. Similar relationship was observed between CK5/6 and Ki-67 index. If we use the 80% cut-off to define the basal/squamous-like phenotype (CK5/6 and GATA3 staining in >80% and ≤80% of tumor cells, respectively), this subgroup would account for 20 (22.0%) cases in our MIBC cohort. Similar to GATA3 alone, the basal/squamous-like cases had a worse RFS (*p* = 0.027) compared with others in the chemotherapy-naïve group. Moreover, CK5/6 alone was not prognostically significant regarding RFS. From the view of prognostication, decrease in GATA3 expression may be a more important component than diffuse CK5/6 staining in the basal/squamous-like phenotype.

Although GATA3 and CK5/6 demosntrated significantly negative correlation in our study, staining for these two markers showed certain overlap in 44 (48.4%) MIBC cases. Furthermore, diffuse coexpression was not rarely encountered (14.29%) in our study. Such a diffuse coexpression phenomenon has not been well-described in previous studies, and simultaneously high GATA3 and CK5/6 expression on protein level were uncommon or even absent in the studies by Sjödahl [34] and Hodgson [19]. Dadhania et al showed some cases with overlap in GATA3 and CK5/6 staining; however, tumors with >80% positivity for both markers were absent in their data [13]. A possible explanation to this phenomenon is the ethnic difference. Further research based on Asian population is warranted to confirm these findings.

The prognostic significance of GATA3 in bladder UC was controversial in previous studies [17–20]. Miyamoto et al reported that loss of GATA3 expression predicted poor prognosis for patients with MIBC [17], but three other studies showed that GATA3 expression had no significant influence on either DSS or RFS [18–20]. In addition to ethnicity, three possible reasons can explain this discrepancy. First, we used whole slides of the tumor specimens for IHC staining instead of tissue microarrays (TMAs), which was the case in previous studies [18–20]. Partial staining might lead to false-negative results in TMA, and this could potentially affect the significance in survival analyses. Second, Kollberg et al included 66 (17%) stage T1 tumors along with MIBC [20], which may influence the results. Finally, in contrast to previous studies [9, 10], the molecular subtypes in their cohort was not associated with clinical outcomes [20]. The potential difference in the underlying population might result in such discrepancy.

As CK20 and CK5/6 are well-established markers related to molecular subtypes [3, 13, 14, 34], it may appear unreasonable that these markers did not have prognostic significance. However, the molecular subtypes were defined through hierarchical analysis with a large panel of markers. Previous studies revealed that the molecular subtypes were associated with clinical outcomes [3, 13, 14, 34], but each marker related to the subtypes was not necessarily significant in clinical outcomes. Kollberg et al also showed no prognostic significance in any single subtype-associated marker [20]. Therefore, the association of GATA3 expression and clinical outcome merits further investigation to prove its value in clinical management.

The interpretation of p53 IHC staining is also noteworthy. Although we could not find the prognostic significance of p53 staining, the differences in the Ki-67 index suggested that using the same criteria as those used for ovarian carcinoma would be more suitable when interpreting p53 staining in bladder cancer. Hodgson et al discovered that null staining of p53 should be considered an aberrant staining pattern [25]. Our study further revealed the potential importance of intensity in the diffuse nuclear staining group. Tumors with diffuse nulcear staining but variable intensity for p53 (score 2) did not have higher Ki-67 indices than did the partial staining (score 1) group. However, the differences in the variable-intensity (score 2) and strong-intensity (score 3) groups were significant. Based on our findings, in a future study, we plan to correlate these criteria with the *TP53* gene status and verify their superiority over the traditional overexpression criteria for bladder cancer.

In conclusion, decrease in GATA3 staining was significantly associated with high proliferative activity and poor clinical outcome in MIBC. IHC staining for GATA3 might facilitate in risk stratification in patients with MIBC receiving radical cystectomy. Combining CK5/6 and GATA3 for prognostic stratification has potential problems because coexpression is common. Defining the “aberrant” p53 staining by using the criteria for ovarian carcinoma (complete absence, strong nuclear reactivity in ≥60% of tumor cells, or diffuse cytoplasmic staining) may be more suitable than the traditional overexpression concept; however, our results did not demonstrate the direct association of p53 expression with clinical outcome.

## Supporting information

S1 Dataset Detailed clinicopathological data of the 91 patients included in this study. (XLSX)

S1 Fig Intratumoral heterogeneity in case RC01-22. The major component demonstrates squamous differentiation with diffuse CK5/6 staining, whereas the minor component is characterized by heavy lymphocytic infiltration and aberrant p53 staining.
(TIF)

S1 Table Immunoreactive score (IRS) by Remmele and Stegner’s criteria. (DOCX)

S2 Table Correlation among GATA3, CK20, and CK5/6 IRS results and Ki-67 index. (DOCX)

S3 Table Cox regression analysis of clinical outcomes in the 78 chemotherapy-naïve patients using tumor staging and IRS of GATA3, CK20, and CK5/6. (DOCX)

S1 Supplementary Data Supplementary analytical data regarding IHC expression of GATA3, CK20, CK5/6, and p53. (XLSX)

## References

[1] National Comprehensive Cancer Network. NCCN Clinical Practice Guidelines in Oncology: Bladder Cancer (Version 1.2019). 10.6004/jnccn.2018.0071 30181422

[2] Netto GJ. Molecular biomarkers in urothelial carcinoma of the bladder: are we there yet? Nat Rev Urol. 2012;9: 41–51. 10.1038/nrurol.2011.193 22158597

[3] Choi W, Porten S, Kim S, Willis D, Plimack ER, Hoffman-Censits J, et al Identification of distinct basal and luminal subtypes of muscle-invasive bladder cancer with different sensitivities to frontline chemotherapy. Cancer Cell. 2014;25: 152–165. 10.1016/j.ccr.2014.01.009 24525232 PMC4011497

[4] Sjödahl G, Lauss M, Lövgren K, Chebil G, Gudjonsson S, Veerla S, et al A molecular taxonomy for urothelial carcinoma. Clin Cancer Res. 2012;18: 3377–3386. 10.1158/1078-0432.CCR-12-0077-T 22553347

[5] Cancer Genome Atlas Research Network. Comprehensive molecular characterization of urothelial bladder carcinoma. Nature. 2014;507: 315–322. 10.1038/nature12965 24476821 PMC3962515

[6] Damrauer JS, Hoadley KA, Chism DD, Fan C, Tiganelli CJ, Wobker SE, et al Intrinsic subtypes of high-grade bladder cancer reflect the hallmarks of breast cancer biology. Proc Natl Acad Sci U S A. 2014;111: 3110–3115. 10.1073/pnas.1318376111 24520177 PMC3939870

[7] Perou CM, Sørlie T, Eisen MB, van de Rijn M, Jeffrey SS, Rees CA, et al Molecular portraits of human breast tumours. Nature. 2000;406: 747–752. 10.1038/35021093 10963602

[8] Sorlie T, Tibshirani R, Parker J, Hastie T, Marron JS, Nobel A, et al Repeated observation of breast tumor subtypes in independent gene expression data sets. Proc Natl Acad Sci U S A. 2003;100: 8418–23. 10.1073/pnas.0932692100 12829800 PMC166244

[9] Robertson AG, Kim J, Al-Ahmadie H, Bellmunt J, Guo G, Cherniack AD, et al Comprehensive Molecular Characterization of Muscle-Invasive Bladder Cancer. Cell. 2017;171: 540–556.e25. 10.1016/j.cell.2017.09.007 28988769 PMC5687509

[10] Kamoun A, de Reyniès A, Allory Y, Sjödahl G, Robertson AG, Seiler R, et al The consensus molecular classification of muscle-invasive bladder cancer. BioRxiv. 2018 12 10. doi: 10.1101/488460. PMC7690647 31563503

[11] Allred C, Miller K, Viale G, Brogi E, Isola J. Molecular testing for estrogen receptor, progesterone receptor, and HER2 In: Lakhani SR, Ellis IO, Schnitt SJ, Tan PH, van de Vijver MJ, editors. WHO classification of tumours of the breast. 4th ed Lyon: IARC; 2012, pp. 22–23.

[12] National Comprehensive Cancer Network. NCCN Clinical Practice Guidelines in Oncology: Breast Cancer (Version 3.2018).

[13] Dadhania V, Zhang M, Zhang L, Bondaruk J, Majewski T, Siefker-Radtke A, et al Meta-analysis of the luminal and basal subtypes of bladder cancer and the identification of signature immunohistochemical markers for clinical use. EBioMedicine. 2016;12: 105–117. 10.1016/j.ebiom.2016.08.036 27612592 PMC5078592

[14] Lerner SP, McConkey DJ, Hoadley KA, Chan KS, Kim WY, Radvanyi F, et al Bladder Cancer Molecular Taxonomy: Summary from a Consensus Meeting. Bladder Cancer. 2016;2: 37–47. 10.3233/BLC-150037 27376123 PMC4927916

[15] Inoue S, Mizushima T, Fujita K, Meliti A, Ide H, Yamaguchi S, et al GATA3 immunohistochemistry in urothelial carcinoma of the upper urinary tract as a urothelial marker and a prognosticator. Hum Pathol. 2017;64: 83–90. 10.1016/j.humpath.2017.04.003 28428106

[16] Sikic D, Keck B, Wach S, Taubert H, Wullich B, Goebell PJ, et al Immunohistochemical subtyping using CK20 and CK5 can identify urothelial carcinomas of the upper urinary tract with a poor prognosis. PLoS One. 2017 6 20;12: e0179602 10.1371/journal.pone.0179602 28632777 PMC5478149

[17] Miyamoto H, Izumi K, Yao JL, Li Y, Yang Q, McMahon LA, et al GATA binding protein 3 is down-regulated in bladder cancer yet strong expression is an independent predictor of poor prognosis in invasive tumor. Hum Pathol. 2012;43: 2033–40. 10.1016/j.humpath.2012.02.011 22607700

[18] Mohammed KH, Siddiqui MT, Cohen C. GATA3 immunohistochemical expression in invasive urothelial carcinoma. Urol Oncol. 2016;34: 432.e9–432.e13. 10.1016/j.urolonc.2016.04.016 27241168

[19] Hodgson A, Liu SK, Vesprini D, Xu B, Downes MR. Basal-subtype bladder tumours show a 'hot' immunophenotype. Histopathology. 2018;73: 748–757. 10.1111/his.13696 29947424

[20] Kollberg P, Chebil G, Eriksson P, Sjödahl G, Liedberg F. Molecular subtypes applied to a population-based modern cystectomy series do not predict cancer-specific survival. Urol Oncol. 2019 5 2 pii: S1078–1439(19)30143–7. 10.1016/j.urolonc.2019.04.010 [Epub ahead of print] 31056435

[21] Esrig D, Spruck CH 3rd, Nichols PW, Chaiwun B, Steven K, Groshen S, et al p53 nuclear protein accumulation correlates with mutations in the p53 gene, tumor grade, and stage in bladder cancer. Am J Pathol. 1993;143: 1389–1397. 7901994 PMC1887166

[22] Finlay CA, Hinds PW, Tan TH, Eliyahu D, Oren M, Levine AJ. Activating mutations for transformation by p53 produce a gene product that forms an hsc70–p53 complex with an altered half-life. Mol Cell Biol. 1988;8: 531–539. 10.1128/mcb.8.2.531 2832726 PMC363177

[23] Kraiss S, Spiess S, Reihsaus E, Montenarh M. Correlation of metabolic stability and altered quaternary structure of oncoprotein p53 with cell transformation. Exp Cell Res. 1991;192: 157–164. 10.1016/0014-4827(91)90170-y 1984409

[24] Malats N, Bustos A, Nascimento CM, Fernandez F, Rivas M, Puente D, et al P53 as a prognostic marker for bladder cancer: a meta-analysis and review. Lancet Oncol. 2005;6: 678–686. 10.1016/S1470-2045(05)70315-6 16129368

[25] Hodgson A, Xu B, Downes MR. P53 immunohistochemistry in high-grade urothelial carcinoma of the bladder is prognostically significant. Histopathology. 2017;71: 296–304. 10.1111/his.13225 28342221

[26] Yemelyanova A, Vang R, Kshirsagar M, Lu D, Marks MA, Shih IeM, et al Immunohistochemical staining patterns of p53 can serve as a surrogate marker for TP53 mutations in ovarian carcinoma: an immunohistochemical and nucleotide sequencing analysis. Mod Pathol. 2011;24: 1248–1253. 10.1038/modpathol.2011.85 21552211

[27] Garg K, Leitao MM Jr, Wynveen CA, Sica GL, Shia J, Shi W, et al P53 overexpression in morphologically ambiguous endometrial carcinomas correlates with adverse clinical outcomes. Mod Pathol. 2010;23: 80–92. 10.1038/modpathol.2009.153 19855378

[28] Remmele W, Stegner HE. Recommendation for uniform definition of an immunoreactive score (IRS) for immunohistochemical estrogen receptor detection (ER-ICA) in breast cancer tissue. Pathol. 1987;8: 138–140. 3303008

[29] Fedchenko N, Reifenrath J. Different approaches for interpretation and reporting of immunohistochemistry analysis results in the bone tissue–a review. Diagn Pathol. 2014; 9: 221 10.1186/s13000-014-0221-9 25432701 PMC4260254

[30] Köbel M, Piskorz AM, Lee S, Lui S, LePage C, Marass F, et al Optimized p53 immunohistochemistry is an accurate predictor of TP53 mutation in ovarian carcinoma. J Pathol Clin Res. 2016;2: 247–258. 10.1002/cjp2.53 27840695 PMC5091634

[31] Dowsett M, Nielsen TO, A’Hern R, Bartlett J, Coombes RC, Cuzick J, et al Assessment of Ki67 in breast cancer: recommendations from the International Ki67 in Breast Cancer Working Group. J Natl Cancer Inst. 2011;103: 1–9. 10.1093/jnci/djr393 PMC3216967 21960707

[32] Liu H, Shi J, Wilkerson ML, Lin F. Immunohistochemical evaluation of GATA3 expression in tumors and normal tissues: a useful immunomarker for breast and urothelial carcinomas. Am J Clin Pathol. 2012;138: 57–64. 10.1309/AJCP5UAFMSA9ZQBZ 22706858

[33] Miettinen M, McCue PA, Sarlomo-Rikala M, Rys J, Czapiewski P, Wazny K, et al GATA3: a multispecific but potentially useful marker in surgical pathology: a systematic analysis of 2500 epithelial and nonepithelial tumors. Am J Surg Pathol. 2014;38: 13–22. 10.1097/PAS.0b013e3182a0218f 24145643 PMC3991431

[34] Sjödahl G, Eriksson P, Liedberg F, Höglund M. Molecular classification of urothelial carcinoma: global mRNA classification versus tumour-cell phenotype classification. J Pathol. 2017;242: 113–125. 10.1002/path.4886 28195647 PMC5413843

