# Supplementary figures and images for: Biological significance of GATA3, cytokeratin 20, cytokeratin 5/6 and p53 expression in muscle-invasive bladder cancer
Source: PLoS One. 2019 Aug 30;14(8):e0221785. doi: 10.1371/journal.pone.0221785 (PMC6716637; doi:10.1371/journal.pone.0221785)

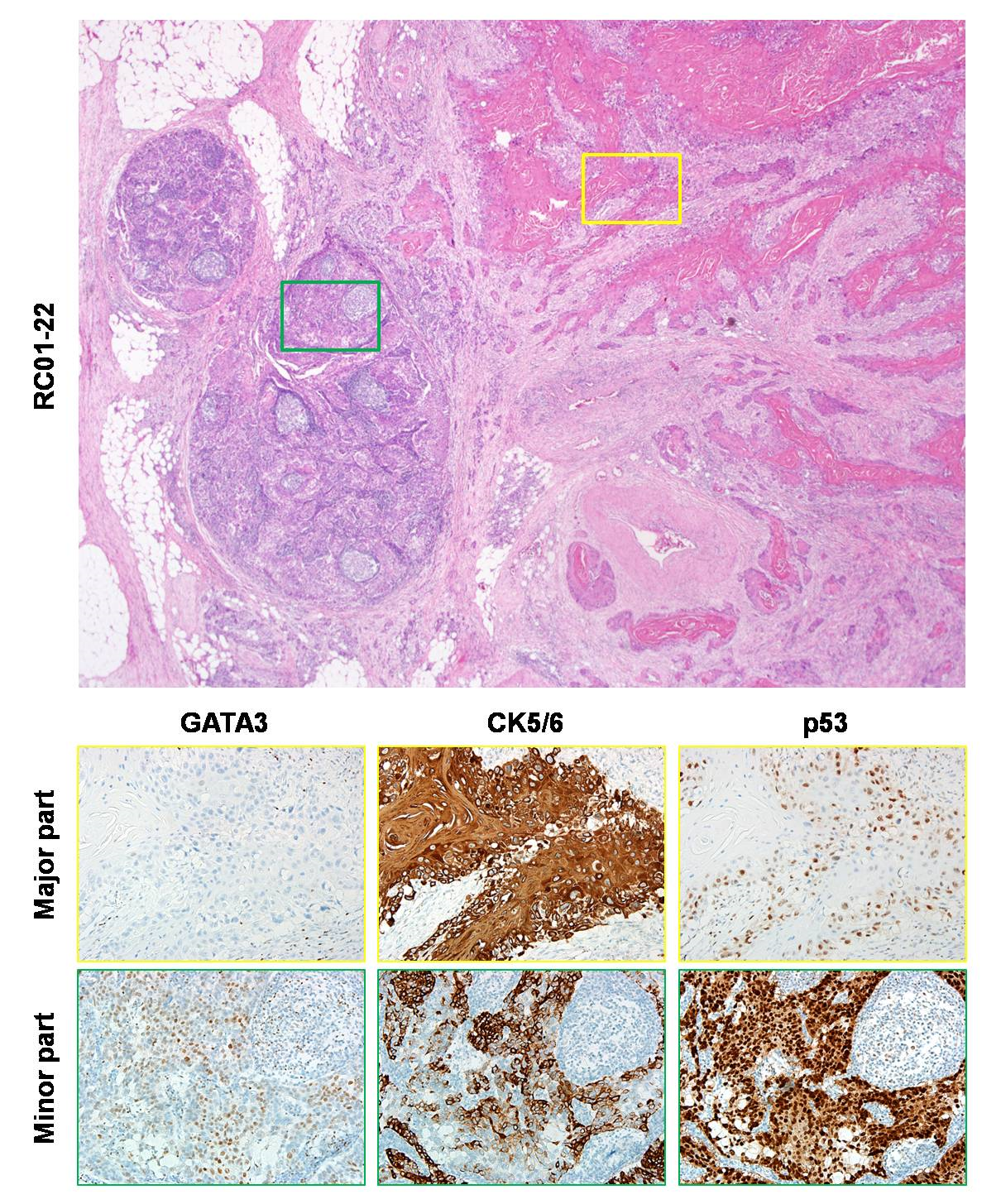

Supplement: S1 Fig — The major component demonstrates squamous differentiation with diffuse CK5/6 staining, whereas the minor component is characterized by heavy lymphocytic infiltration and aberrant p53 staining. (TIF) [file pone.0221785.s002.tif]
