# Supplementary material for: Biological significance of GATA3, cytokeratin 20, cytokeratin 5/6 and p53 expression in muscle-invasive bladder cancer
Source: PLoS One. 2019 Aug 30;14(8):e0221785. doi: 10.1371/journal.pone.0221785 (PMC6716637; doi:10.1371/journal.pone.0221785)
Supplement: S1 Table — (DOCX) [file pone.0221785.s003.docx]

**S1 Table. Immunoreactive score (IRS) by Remmele and Stegner’s criteria.**

| **A**: Percentage of positive cells | **B**: Intensity of staining |
| --- | --- |
| **0**: no positive cells | **0**: no color reaction |
| **1**: <10% positive cells | **1**: mild reaction |
| **2**: 10–50% positive cells | **2**: moderate reaction |
| **3**: 51–80% positive cells | **3**: intense reaction |
| **4**: >80% positive cells |  |
| **Final IRS score (A × B): 0–12** | |
