# Supplementary material for: Biological significance of GATA3, cytokeratin 20, cytokeratin 5/6 and p53 expression in muscle-invasive bladder cancer
Source: PLoS One. 2019 Aug 30;14(8):e0221785. doi: 10.1371/journal.pone.0221785 (PMC6716637; doi:10.1371/journal.pone.0221785)
Supplement: S2 Table — (DOCX) [file pone.0221785.s004.docx]

**S2 Table. Correlation among IRS results of GATA3, CK20, CK5/6 and Ki-67 index.**

|  | Correlation in IRS^a^ (95% CI) | | | |
| --- | --- | --- | --- | --- |
|  | GATA3 | CK20 | CK5/6 | Ki-67 |
| GATA3 | 1.000 | 0.549 (0.381 – 0.682) | 0.413 (-0.574 – -0.220) | -0.333 (-0.509 – -0.130) |
| CK20 |  | 1.000 | -0.462 (-0.614 – -0.278) | -0.371 (-0.541 – -0.173) |
| CK5/6 |  |  | 1.000 | 0.244 (0.034 – 0.433) |
| Ki-67 |  |  |  | 1.000 |

Abbreviation: IRS, immunoreactive score; CI, confidence interval

^a^Applies to GATA3, CK20 and CK5/6.
