# Supplementary material for: Biological significance of GATA3, cytokeratin 20, cytokeratin 5/6 and p53 expression in muscle-invasive bladder cancer
Source: PLoS One. 2019 Aug 30;14(8):e0221785. doi: 10.1371/journal.pone.0221785 (PMC6716637; doi:10.1371/journal.pone.0221785)
Supplement: S3 Table — (DOCX) [file pone.0221785.s005.docx]

**S3 Table. Cox regression analysis of clinical outcomes in the 78 chemotherapy-naïve patients using tumor staging and IRS of GATA3, CK20 and CK5/6.**

| Variable | Univariate |  | Multivariate |  |
| --- | --- | --- | --- | --- |
|  | HR (95% CI) | *p* value | HR (95% CI) | *p* value |
| **RFS** |  |  |  |  |
| T stage (T2 reference) | 1.837 (1.230–2.745) | 0.003 | 1.639 (1.053–2.551) | 0.029 |
| Nodal metastasis (N1–3 vs. N0) | 1.944 (1.091–3.463) | 0.024 | 1.806 (0.962–3.391) | 0.065 |
| GATA3(by IRS) | 0.911 (0.849–0.977) | 0.010 | 0.897 (0.834–0.965) | 0.004 |
| CK20 (by IRS) | 0.963 (0.905–1.025) | 0.238 |  |  |
| CK5/6 (by IRS) | 1.034 (0.973–1.098) | 0.283 |  |  |
| **DSS** |  |  |  |  |
| T stage (T2 reference) | 1.343 (0.786–2.294) | 0.281 |  |  |
| Nodal metastasis (N1–3 vs. N0) | 2.257 (1.059–4.811) | 0.035 |  |  |
| GATA3 (by IRS) | 0.924 (0.844–1.013) | 0.091 |  |  |
| CK20 (by IRS) | 0.967 (0.889–1.052) | 0.438 |  |  |
| CK5/6 (by IRS) | 1.042 (0.963–1.127) | 0.309 |  |  |
| **OS** |  |  |  |  |
| T stage (T2 reference) | 1.460 (0.887–2.403) | 0.136 |  |  |
| Nodal metastasis (N1–3 vs. N0) | 1.702 (0.838–3.457) | 0.142 |  |  |
| GATA3 (by IRS) | 0.934 (0.858–1.017) | 0.117 |  |  |
| CK20 (by IRS) | 0.956 (0.882–1.036) | 0.270 |  |  |
| CK5/6 (by IRS) | 1.047 (0.973–1.126) | 0.222 |  |  |

Abbreviations: HR, hazard ratio; CI, confidence interval
